# A pan-cancer analysis of anti-proliferative protein family genes for therapeutic targets in cancer

**DOI:** 10.1038/s41598-023-48961-1

**Published:** 2023-12-07

**Authors:** Siming Zhang, Jue Gu, Ling-ling Shi, Bo Qian, Xun Diao, Xiaohui Jiang, Jindong Wu, Zhijun Wu, Aiguo Shen

**Affiliations:** 1 Cancer Research Center Nantong, Nantong Tumor Hospital and Affiliated Tumor Hospital of Nantong University, Nantong, Jiangsu China; 2 Affiliated Hospital of Nantong University, Nantong, China; 3 Affiliated Nantong Hospital Third of Nantong University, Nantong, China; 4 Maternal and Child Care Hospital of Qidong, Nantong, China; 5 Department of General Surgery, Nantong Tumor Hospital and Affiliated Tumor Hospital of Nantong University, Nantong, China; 6 Department of Oncology, Nantong Traditional Chinese Medicine Hospital, Nantong, China

**Keywords:** Cancer, Tumour biomarkers, Computational biology and bioinformatics, Databases, Bioinformatics

## Abstract

The recently discovered APRO (anti-proliferative protein) family encodes a group of trans-membrane glycoproteins and includes 6 members: TOB1, TOB2, BTG1, BTG2, BTG3 and BTG4. The APRO family is reportedly associated with the initiation and progression of cancers. This study aims to undertake a comprehensive investigation of the APRO family of proteins as a prognostic biomarker in various human tumors. We performed a pan-cancer analysis of the APRO family based on The Cancer Genome Atlas (TCGA). With the bioinformatics methods, we explored the prognostic value of the APRO family and the correlation between APRO family expression and tumor mutation burden (TMB), microsatellite instability (MSI), drug sensitivity, and immunotherapy in numerous cancers. Our results show that the APRO family was primarily down-regulated in cancer samples. The expression of APRO family members was linked with patient prognosis. In addition, APRO family genes showed significant association with immune infiltrate subtypes, tumor microenvironment, and tumor cell stemness. Finally, our study also demonstrated the relationship between APRO family genes and drug sensitivity. This study provides comprehensive information to understand the APRO family’s role as an oncogene and predictor of survival in some tumor types.

## Introduction

The APRO (anti-proliferative protein) family includes 6 members: TOB1, TOB2, BTG1, BTG2, BTG3 and BTG4 which not only involve in the regulation of cell growth and development^1^, but also play an important role in apoptosis, invasion and metastasis of various tumors^2^, and some members are of great significance for the prognosis of tumors^3–6^.

Tumor suppressor gene TOB1 is mainly participated in the tumor occurrence as well as T cell activation^7, 8^. Bai et al. found that TOB1 could inhibit the proliferation of malignant pancreatic cells^9^. Therefore, some researchers believed that TOB1 could be used as an independent indicator to evaluate the prognosis of patients with gastric cancer (GC)^10, 11^. For instance, in the study of esophageal squamous cell carcinoma (ESCC), down-regulated TOB1 expression was found to be correlated with the unfavorable prognosis of tumor patients^12^. Additionally, TOB1 was also uncovered to trigger autophagy to repress GC progression through activating AKT/mTOR pathway^10^. BTG1 mutations may disrupt a critical immune gatekeeper mechanism that strictly limits B cell fitness during antibody affinity maturation in diffuse large B-cell lymphoma^13, 14^. The overexpressed BTG2 could decrease the proliferation and migration of glioma cells^15^ and may act as an effective target for the treatment of luminal A breast cancer^16^. As a result, BTG1 and BTG2 are considered as tumor suppressors and closely correlated with tumor cell behavior as well as prognosis^17^. An et al. suggested that BTG3 overexpression could inhibit cell proliferation and invasion and promote cell apoptosis in epithelial ovarian cancer by regulating the AKT/GSK3β/β-catenin signaling pathway^18^.

In the present study, we comprehensively analyzed the role of TOB1, TOB2, BTG1, BTG2, BTG3 and BTG4 in cancer from the perspective of bioinformatics. We intended to disclose correlations between the expression of the APRO family and clinical features, prognosis, and tumor-infiltrating immune cells. In addition, we also aimed to demonstrate the expression of the APRO family changes during the immune response, tumor microenvironment, tumor stemness, and cancer cell sensitivity. Through a series of bioinformatics analysis, the findings in our study supported the important role of TOB1, TOB2, BTG1, BTG2, BTG3 and BTG4 in pan-cancer and provided a reliable basis for detecting biomarkers of cancer.

## Methods

### Data collection

**Figure 1:**
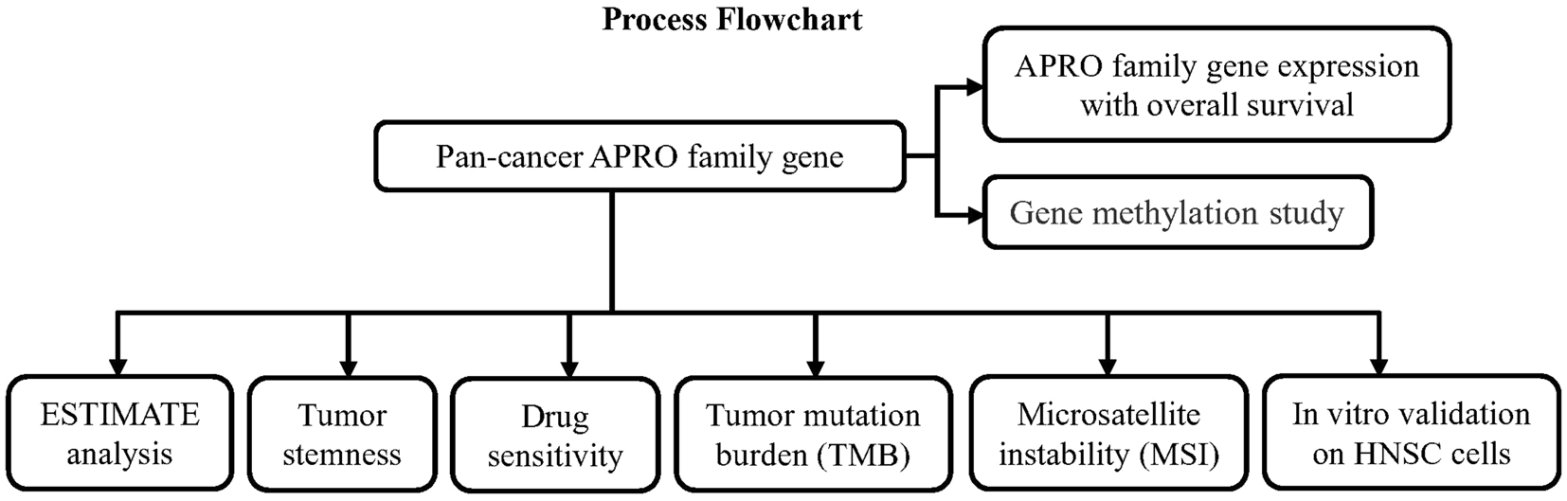
The study flow chart.

The analysis process of our study is shown in Fig. 1. We obtained pan-cancer from TCGA database combined with transcriptome data and clinical information from a public database UCSC Cancer Genomics Browser (https://xenabrowser.net/datapages/). The Imvigor210 cohort was downloaded from the website based on the Creative Commons 3.0 license (http://research-pub.Gene.com/imvigor210corebiologies).

### Pan-cancer analysis of APRO family genes expression

Preliminarily, we drew boxplots of APRO family genes expression within general pan-cancers or single cancer to test the difference in gene expression distribution. Then, heatmaps were constructed using the R package “pheatmap” (https://CRAN.R-project.org/package=pheatmap) to observe the APRO family genes (TOB1, TOB2, BTG1, BTG2, BTG3 and BTG4) expression between tumors and adjacent normal tissues. Spearman correlation analysis was conducted to detect the expression correlation across APRO family genes. The position of these genes in chromosome was plotted by RCircos package^19^. Additionally, we applied a STRING analysis for protein–protein interaction (PPI) network among APRO family genes. As mutually supplementary analysis, we also input APRO family genes in the “Gene_DE” module of TIMER2 (tumor immune estimation resource, version 2) web (http://timer.cistrome.org/) to detect the expression difference of these genes between tumor and adjacent normal tissues of the TCGA project as Cui et al. described^20^.

### Survival prognosis analysis

We explored the overall survival (OS) map data of the APRO family (TOB1, TOB2, BTG1, BTG2, BTG3 and BTG4) to confirm the prognostic value of APRO family members according to the univariate Cox regression. Then, the patients were divided into a high group and a low group according to the median expression level of APRO family genes. The Kaplan–Meier method and survival rate R package were used to analyze the prognosis of the patients in the 2 groups.

### Methylation analysis

The detailed description on DNA methylation analysis could be seen in published paper^21^. Briefly, GSCALite (bioinfo.life.hust.edu.cn/web/GSCALite/)^22^ website was applied to acquire relevant methylation information of APRO family genes. Here, 14 cancer types containing cancer and counterpart tissues was employed for methylation analysis. Additionally, MethSurv (biit.cs.ut.ee/methsurv/)^23^ online analysis were carried out to precisely investigate the effect of methylation sites.

### Tumor microenvironment infiltration analysis

Six immune subtypes retrieved from a previous reference^24^ were adopted to measure immune infiltrates in tumor environment. Additionally, ESTIMATE as an algorithm produces 3 scores: the StromalScore, ImmuneScore, and ESTIMATEScore^25^. Here, we assessed the association among the expression level of TOB1, TOB2, BTG1, BTG2, BTG3 and BTG4 genes and three ESTIMATE scores via Spearman analysis.

### Stemness scores and drug sensitivity analysis

We also evaluated the association among the expression level of APRO family genes and tumor stemness score (RNAss and DNAss) in substantial tumor data according to Spearman analysis. Next, CellMiner (https://discover.nci.nih.gov/cellminer/) is a web-based application that provides data and a pharmacological overview of the NCI-60 cancer cell line, from which we mined these data^26–28^. To explore the correlation between APRO family genes and drug sensitivity, we downloaded the APRO family gene and compound activity data using the R package. For a supplement, Connectivity Map (CMap, https://clue.io/) website is also implemented for drug sensitivity prediction^29^.

### Tumor mutation burden (TMB) and microsatellite instability analysis

TMB is the sum of mutations per megabase in tumor tissue. Microsatellite instability (MSI) is a hypermutation pattern that occurs on genomic microsatellites and is caused by defects in mismatch repair systems. To evaluate the correlation between APRO family (TOB1, TOB2, BTG1, BTG2, BTG3 and BTG4) expression and TMB/MSI in pan-cancer, we used the dataset comprising mRNA-seq data from TCGA and plotted the resultant figures using the R package “ggstatsplot”.

### Quantitative reverse transcription-polymerase chain reaction (qRT-PCR)

Total RNA was extracted with TRIZOL reagent from the normal epithelial cell line NHOK (Chinese Academy of Sciences of Shanghai) and head and neck squamous cell carcinoma (HNSC) CAL-27 (ATCC: CRL-2095) cell lines. RNA quantity was determined by spectrophotometer. Quantitative reverse transcription-polymerase chain reaction (qRT-PCR) was applied to assess APRO family gene level adopting SYBR Green (Bio-Rad, Hercules, CA). Utilizing the 2^−ΔΔCT^ method, data from the threshold cycle^14^ were obtained and standardized to the levels of GAPDH in each sample. The primers were listed in Table 1.

**Table 1:** The sequences of primer pairs for target genes.

Gene	Forward primer sequence (5′–3′)	Reverse primer sequence (5′–3′)
TOB1	TCTGTATGGGCTTGGCTTG	TGTTGCTGCTGTGGTGGT
TOB2	ATGCAGCTAGAGATCAAAGTGGC	CCAATGTGAACACAGCGGAAG
BTG1	CCACCATGATAGGCGAGATCG	GGTTGATGCGAATACAACGGTA
BTG2	CCTGTGGGTGGACCCCTAT	GGCCTCCTCGTACAAGACG
BTG3	ATGAAATTGCTGCCGTTGTCT	GCCTGTCCTTTCGATGGTTTT
BTG4	AGAAAAGCTGATGACGATCTTGT	TGAAGGCTTGCCCTTTAGAAG
GAPDH	GTCTCCTCTGACTTCAACAGCG	ACCACCCTGTTGCTGTAGCCAA

### Statistical analysis

The expression level of the APRO family was shown, and the derived expressions were evaluated using Wilcoxon tests. The Pearson correlation analysis was used to explore the expression correlation between the APRO family and patient clinical characteristics and immune subtypes. We tested the correlation between gene expression and stemness scores, StromalScore, ImmuneScore, ESTIMATEScore, and drug sensitivity using R software and the following R packages: ggpubr, pheatmap, ggplot2, survminer, and corrplot. P values < 0.05 were considered statistically significant.

## Results

### Pan-cancer APRO family member expression analysis

**Figure 2:**
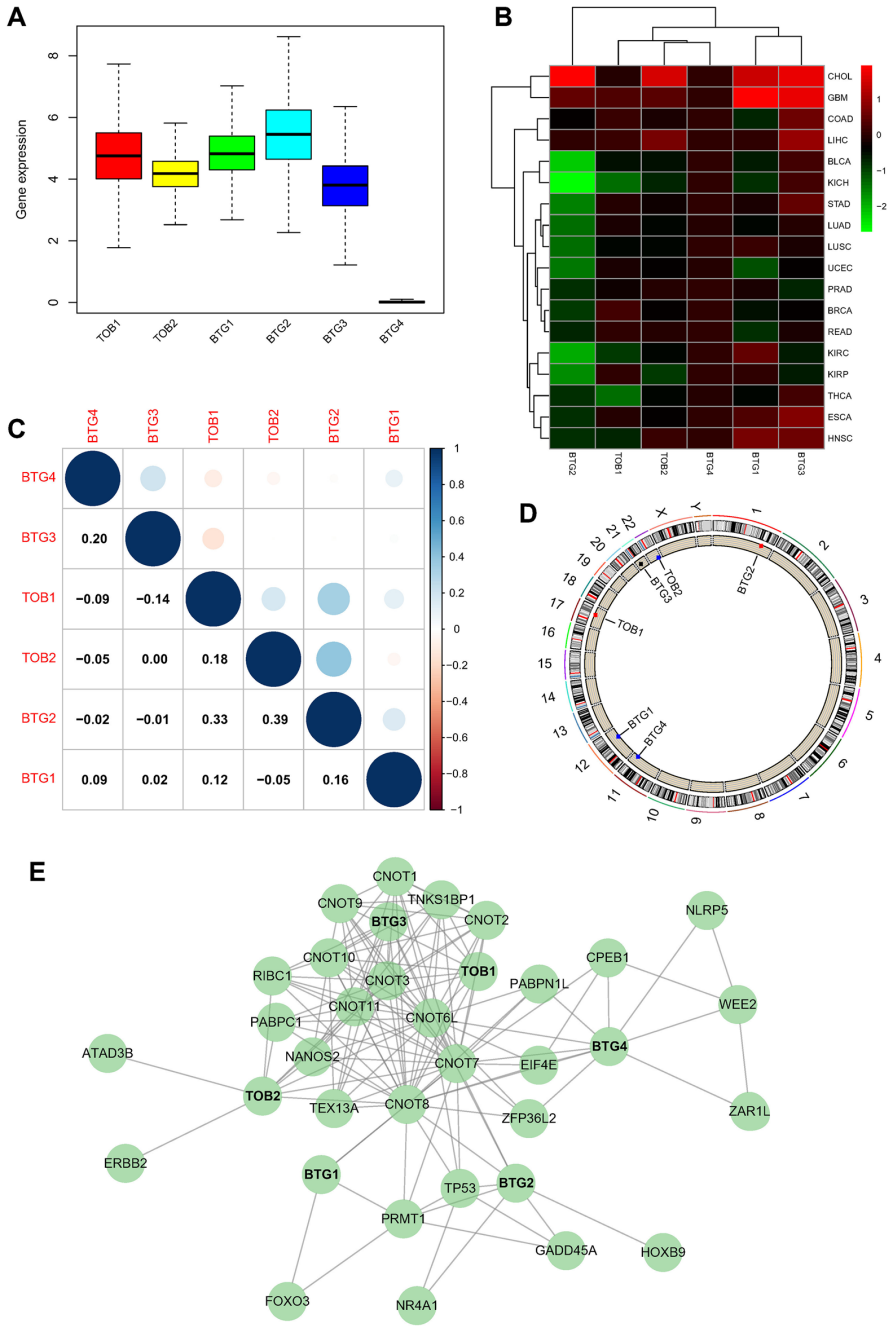
Relevant analysis of the APRO family genes. (**A**) The general expression of APRO family genes within 33 tumor types; (**B**) heatmaps of APRO family gene expression between cancer and counterpart tissues based on log2(fold change) for 18 cancer types which have over 5 counterpart samples (red means high expression, green means low expression, and color depth implies the degree of expression difference); (**C**) correlationship within APRO family genes expression among 33 cancer types; (**D**) alterations of APRO family genes with CNVs on the chromosome; (E) STRING analysis of all APRO genes with other related genes.

To begin, we analyzed the expression of the APRO family in the pan-cancer TCGA database. The analysis is shown in Fig. 2A. The Wilcoxon signed-rank test was used to analyze the differential expression of APRO family genes in cancer and adjacent tissues (Fig. 2B).

In the Fig. 2C, we also explored the association among TOB1, TOB2, BTG1, BTG2, BTG3 and BTG4 and found that BTG3 and BTG4 (r = 0.22), TOB2 and BTG2 (r = 0.39), TOB2 and TOB1(r = 0.18), BTG1 and BTG2 (r = 0.16) display a positive association, but TOB1 had a negative association with BTG4 (r =  − 0.09) and BTG3 (r =  − 0.14), hinting that they possibly have common roles or functions. We also observed the alterations of these genes with CNVs on the chromosome (Fig. 2D) STRING analysis revealed that all APRO family genes seemed independent, as they did not interact with each other, implying an independent role for each gene (Fig. 2E).

### Association of APRO family gene expression and overall survival

**Figure 3:**
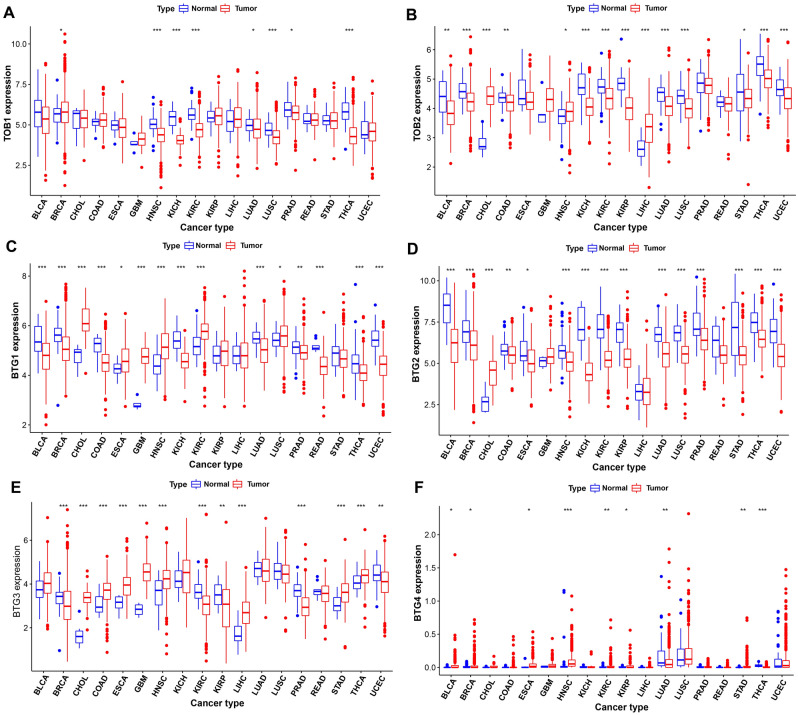
APRO family gene expression differed in certain cancer and normal tissues (**A**) TOB1; (**B**) TOB2; (**C**) BTG1; (**D**) BTG2; (**E**) BTG3; (**F**) BTG4 (***P < 0.001; **P < 0.01; *P < 0.05).

The expression of TOB1 in tumor tissues distinctly differed from that in normal tissues with higher expression levels in breast invasive carcinoma (BRCA), while lower expression levels in HNSC, kidney chromophobe (KICH), kidney renal clear cell carcinoma (KIRC), lung adenocarcinoma (LUAD), lung squamous cell carcinoma (LUSC), prostate adenocarcinoma (PRAD) and thyroid carcinoma (THCA) (Fig. 3A). As shown in Fig. 3B, the expression of TOB2 was lower in tumor tissue than most of normal tissue, including bladder urothelial carcinoma (BLCA), BRCA, colon adenocarcinoma (COAD), kidney chromophobe (KICH), KIRC, kidney renal papillary cell carcinoma (KIRP), LUAD, LUSC, stomach adenocarcinoma (STAD), THCA and uterine corpus endometrial carcinoma (UCEC), except for opposite tendencies in cholangiocarcinoma^30^, HNSC and LIHC. Additionally, Fig. 3C shows the expression of BTG1 was higher in tumor tissue than in normal tissue, including for BLCA, BRCA, COAD, KICH, LUAD, PRAD, READ, THCA, and UCEC. In the CHOL, esophageal carcinoma (ESCA), glioblastoma multiforme (GBM), HNSC, KIRC and LUSC, BTG1 expression was higher in tumor tissue than in normal tissue (all P values < 0.05). In the Fig. 3D,E, the expression levels of BTG2 and BTG3 displayed similar trend towards BTG1. However, the expression levels of BTG4 in tumor was basically low (Fig. 3F). Another TIMER2 analysis revealed similar expression tendency (Supplementary Figure S2). These observations implied the necessities of researching every gene as a whole.

**Figure 4:**
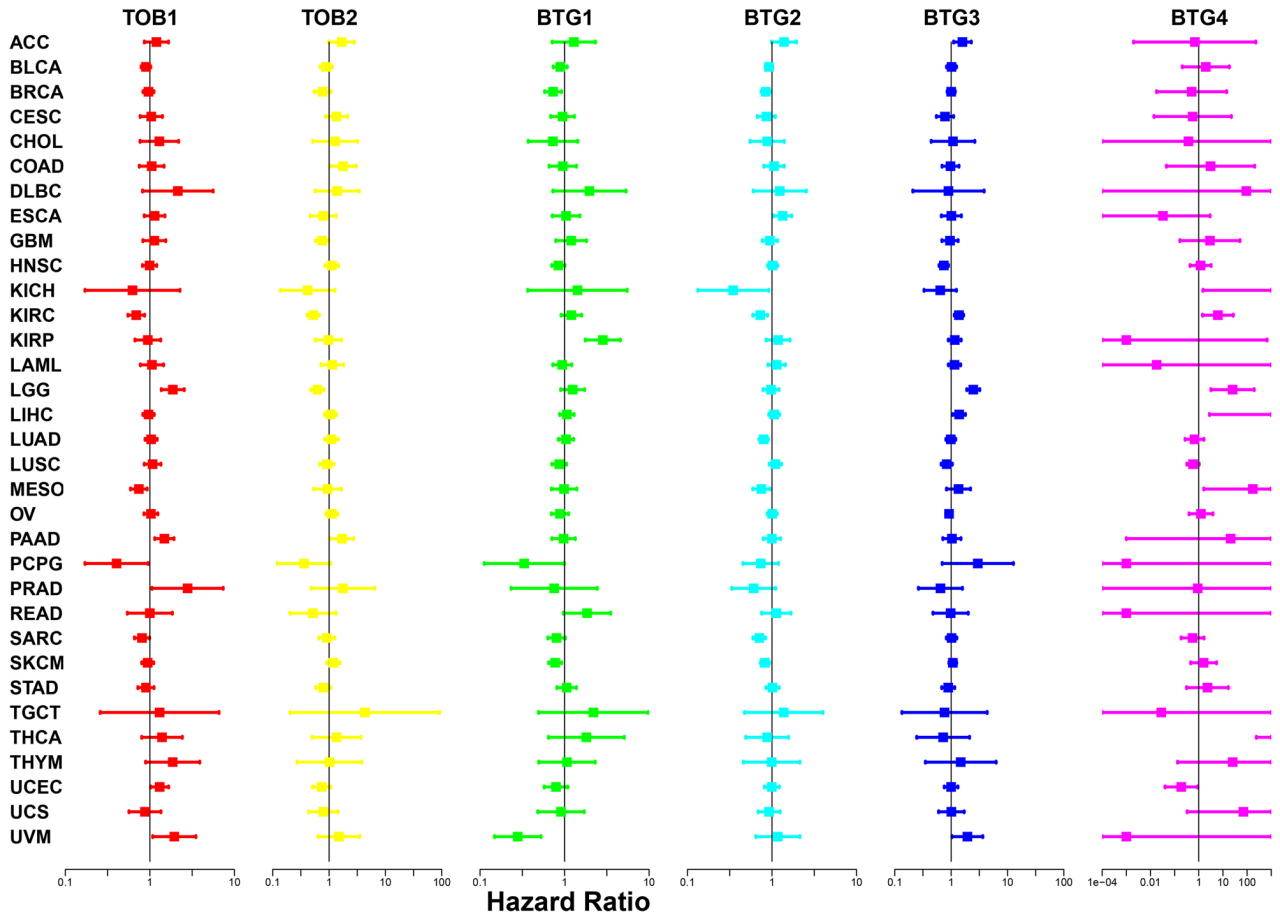
Univariate Cox regression analysis the prognosis of APRO family member across 33 cancer types.

In order to further assess the correlations and predict which APRO members promote or inhibit which kind of cancer types, a univariate Cox proportional hazard regression model was used for this analysis. We found that the change of APRO expression was usually related to the total survival period of patients; however, the correlation differed according to the type of cancer detected. For example, TOB1 predicted the prognosis in KIRC, LGG, MESO, PAAD, PCPG, PRAD, SARC, UCEC and UVM patients, and TOB2 predicted poor prognosis in COAD, GBM, KIRC, LGG, PAAD patients. Besides, BTG1 predicted the prognosis in BRCA, KIRP, PCPG, SKCM, UVM patients. BTG2 predicted survival advantage for BRCA, KICH, KIRC, LUAD, MESO, SARC and SKCM (HR < 1), yet poor prognosis for ESCA (HR > 1). BTG3 was primary associated with poor survival for patients with ACC, KIRC, LGG, LIHC and UVM. BTG4, which was commonly low expressed in cancer and normal tissues, also could predict unfavorable prognosis for KICH, KIRC, LGG, LIHC, MESO and THCA with big HR values, indicating its significance in disease prognosis. Interestingly, we detected that APRO family but BTG1, all could predict the prognosis for KIRC. Four APRO family (TOB1, TOB2, BTG3 and BTG4) could help forecast the prognosis for LGG (Fig. 4; Table S1). As a supplementary analysis, Kaplan–Meier survival analysis further showed that the low expression of TOB1 in KIRC, LIHC, PCGC, SARC and the high expression of TOB1 in LGG, PAAD, PRAD were associated with poor prognosis. More specifically, the high expression of TOB2 in KIRC, PCGC and UCS indicated a favorable prognosis. Increased expression of BTG1 and BTG2 was mainly associated with increased survival advantage, where BTG1 predicted better prognosis of patients with BRCA, HNSC, LUSC, SARC, UVM, and BTG2 predicted better prognosis for BLCA, LUAD, MESO, SARC, SKCM. However, the BTG3 and BTG4 increased mainly correlated with survival disadvantages, where BTG3 predicted poor prognosis for ACC, HNSC, LGG, and BTG4 predicted poor prognosis for HNSC, KIRC, MESO, UCS (Supplement Fig. 1; Table S2).

### DNA methylation analysis

**Figure 5:**
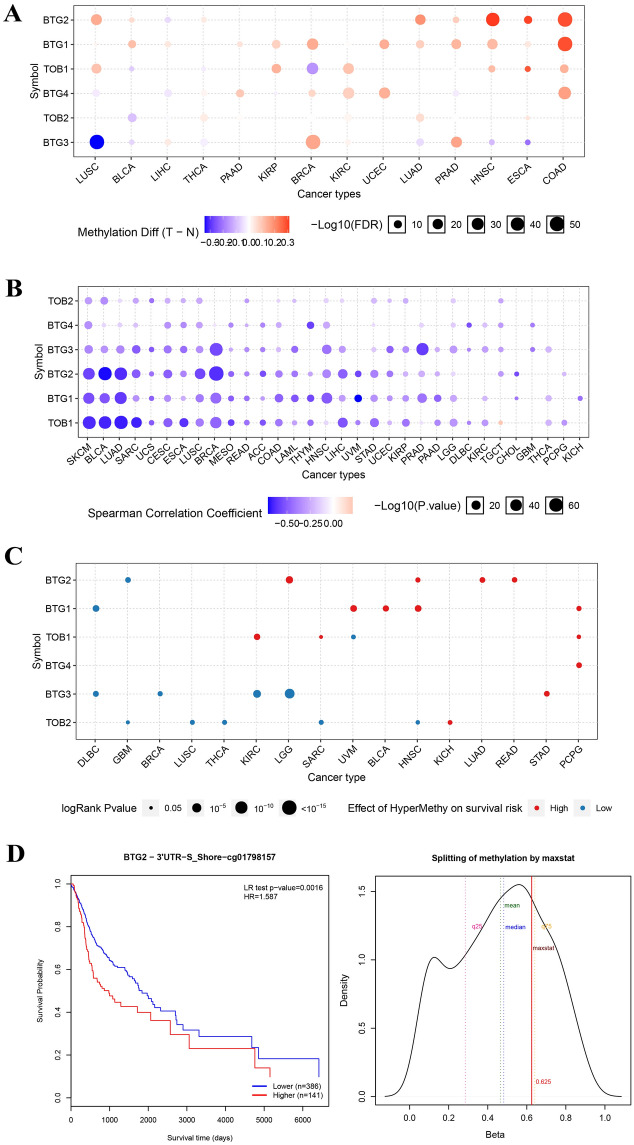
Methylation analysis (**A**) Methylation difference between tumor and normal samples. (**B**) Spearman correlation coefficient of methylation and gene expression. (**C**) Overall survival difference between hypermethylation and hypomethylation. (**D**) Survival analysis and distribution of the methylation level of the BTG2 probes.

The methylation level of some APRO family genes distinctly differed between the cancer and para-cancerous tissues. We could see that the methylation level of BTG2 increased significantly in HNSC, ESCA, and COAD. Meanwhile, BTG2 methylation was noticeably enhanced in LUSC (Fig. 5A). What’s more, an intense and negative relation was observed within methylation degree and APRO family genes expression (Fig. 5B). Additionally, survival risk analysis revealed a consistent trend of high methylation and high risk BTG2 in HNSC (Fig. 5C). In combination with transcriptome analyses that the expression of BTG2 was inhibited in HNSC (Fig. 3D), possibly due to increased methylation, which may account for tumorigenesis and poor prognosis of HNSC. Additionally, we found detailed best methylation sites, cg01798157, which showed an inferior prognosis in the hypermethylation groups for BTG2 in HNSC though (Fig. 5D).

### APRO family genes were associated with immune response and the tumor microenvironment in cancer

**Figure 6:**
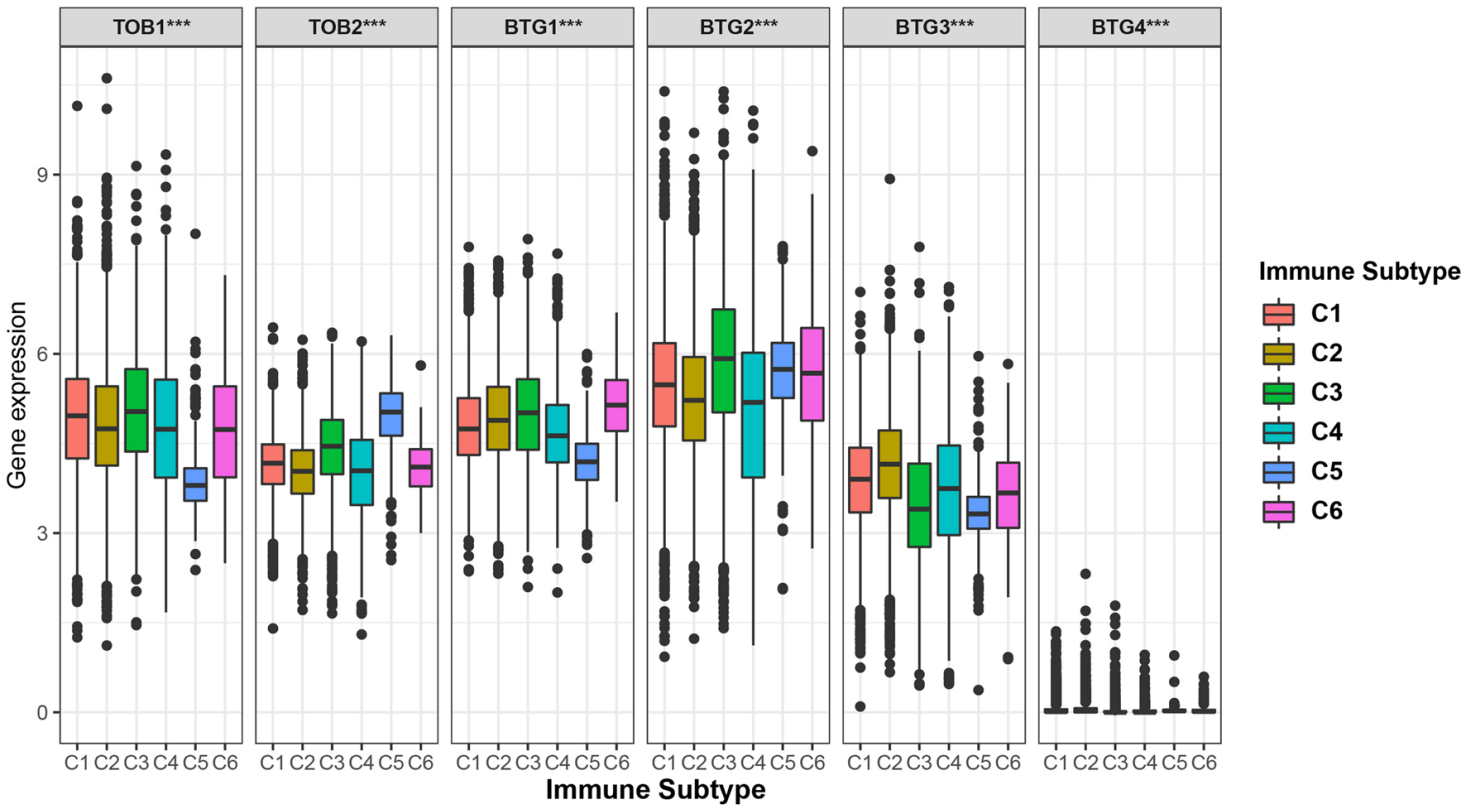
APRO family genes expression analysis in different immune subtypes.

**Figure 7:**
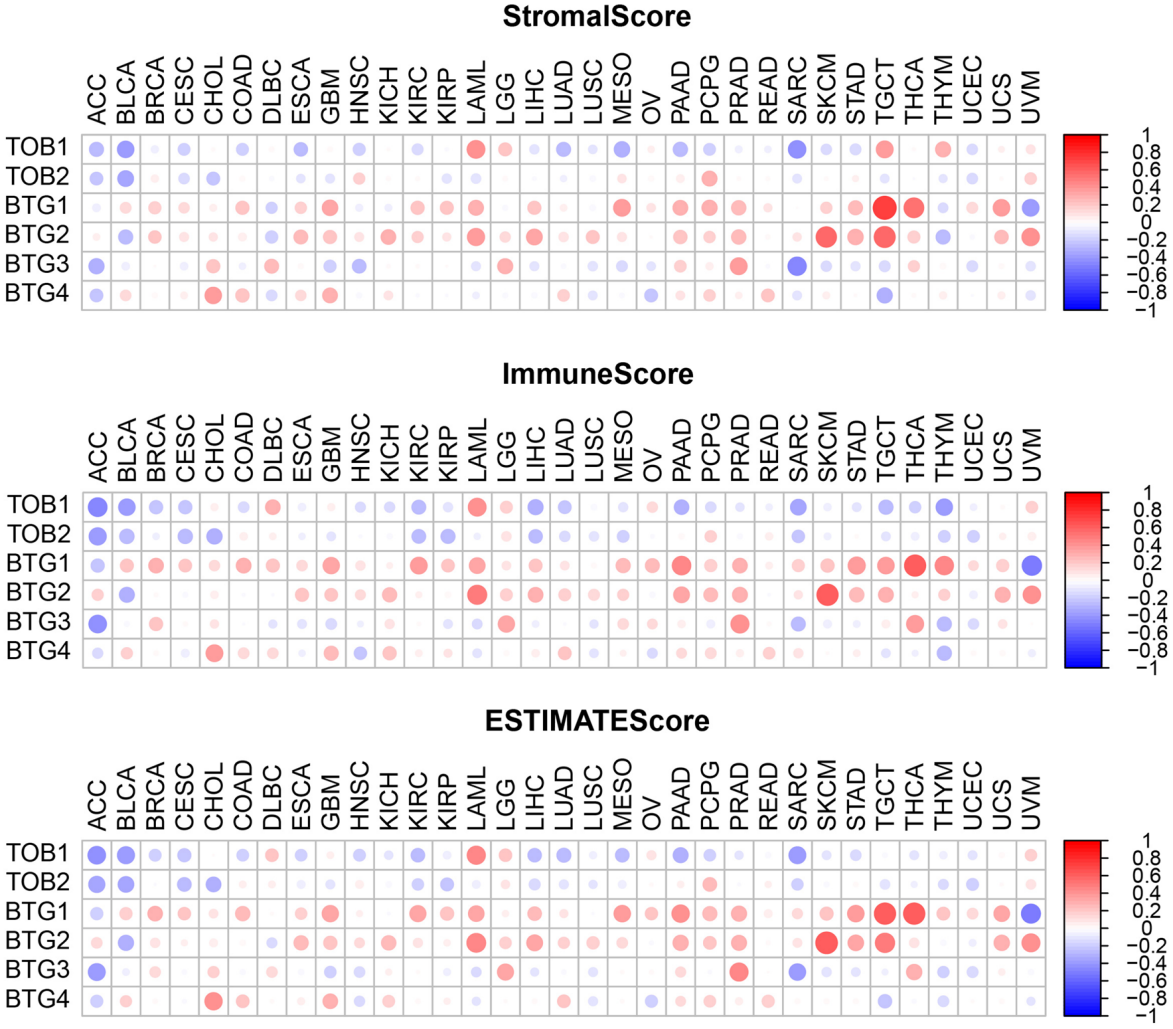
The relationship of APRO family gene expression levels with the StromalScore (**A**), ImmuneScore (**B**), and ESTIMATEScore (**C**).

In order to understand the expression of the APRO family in different immune subtypes, we explored their differential expression. The distribution of immune subtypes in pan-cancer is shown in Table S3. We observed significant differences in APRO family genes among different immune subtypes, with P values less than 0.001 (Fig. 6), suggesting that the APRO family was related to tumor immunity, and the expression level of the BTG2 ranks first in the overall immune subtypes of C1–C6. In addition, we found a close correlation between the APRO family genes and StromalScore in most tumors (Fig. 7A). We found that TOB1 was negatively associated with BLCA, COAD, HNSC, KIRC, LUAD, PAAD, SARC and UCEC. However, TOB1 was positively related to the LAML, LGG, TGCT and THYM. More specifically, we found significant positive correlations between TOB2 and stromal score in HNSC and PCPG, BTG1 in BRCA, COAD, GBM, KIRC, KIRP, LAML, LIHC, MESO, PAAD, PCPG, PRAD, SKCM, STAD, TGCT, THCA, UCEC, BTG2 in BRCA, KIRC, LAML, LIHC, LUSC, PRAD, SKCM, STAD, TGCT, THCA and UVM, BTG3 in LGG, PRAD and THCA, and BTG4 in COAD, GBM and LUAD (P < 0.001). Furthermore, we also analyzed the relevance of the APRO family genes to ImmuneScore (Fig. 7B) and ESTIMATEScore (Fig. 7C) with similar findings.

### APRO family was associated with tumor stemness and cancer cell sensitivity to chemotherapy

**Figure 8:**
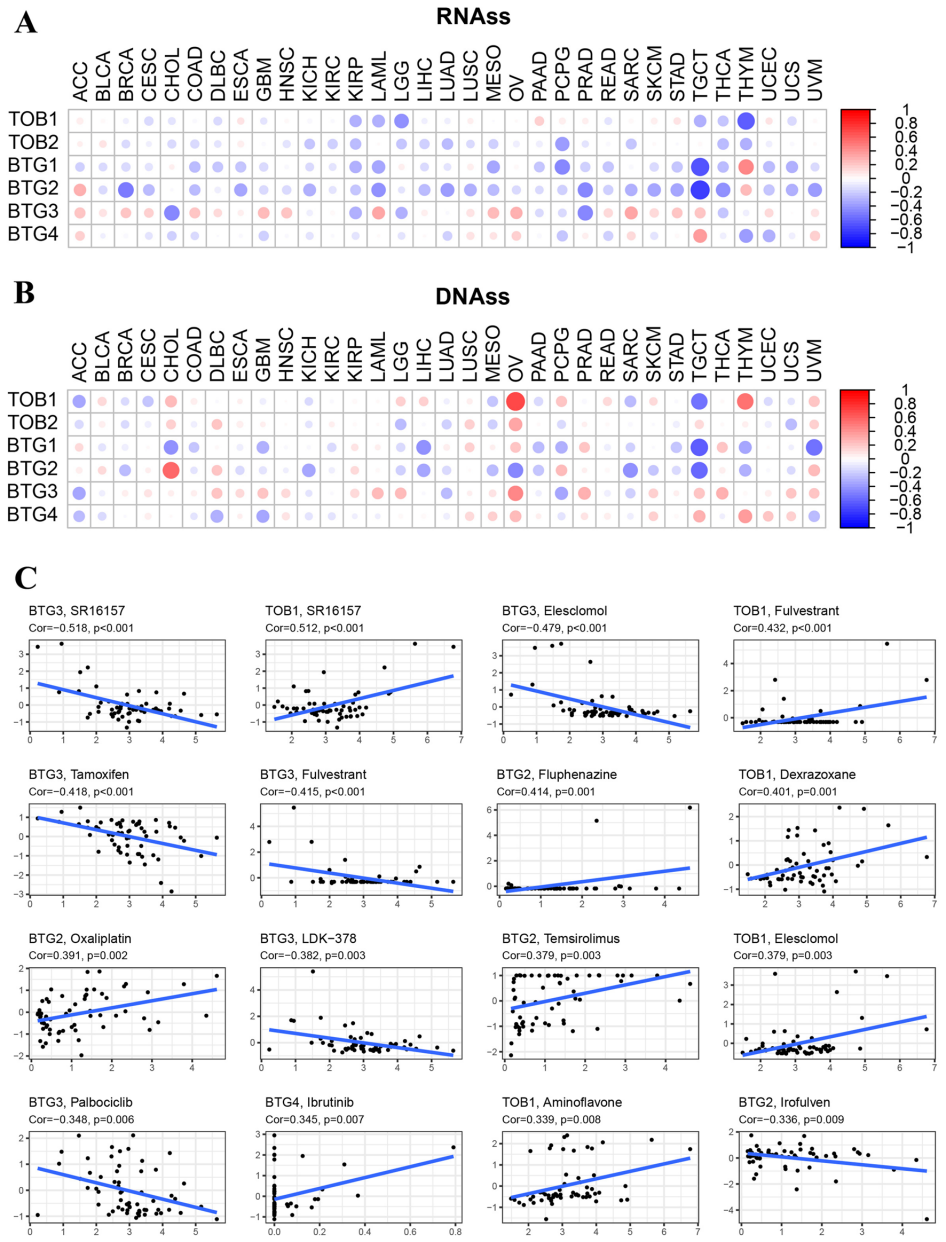
The relationship of APRO family genes expression with tRNAss, DNAss, and drug sensitivity. (**A**,**B**) The correlation between APRO family genes and RNAss and DNAss; (**C**) the correlation between the APRO family and drug sensitivity.

We assessed the associations across the expression level of TOB1, TOB2, BTG1, BTG2, BTG3 and BTG4, within RNAss, DNAss, and drug sensitivity (Fig. 8). APRO family genes had different degrees of correlation with RNAss and DNAss in different cancers. In terms of RNAss, KIRP had a negatively significant correlation with APRO family genes (Fig. 8A). In aspect of DNAss, OV was positively correlated with APRO family excluding BTG2 (Fig. 8B).

Next, we studied the correlation between APRO family gene expression and drug compound activity. We found that the high expression of APRO family genes, especially TOB1 and BTG2, were related to the increased drug sensitivity of many antitumor drugs, however, BTG3 displayed the negatively association with antitumor drugs, suggesting potentials to drug resistance (Fig. 8C, Table 2). For example, TOB1 was positively correlated with drug sensitivity to SR16157, Fulvestrant, Dexrazoxane, Elesclomol and Aminoflavone. BTG2 was associated with drug sensitivity to Fluphenazine, Oxaliplatin and Temsirolimus. Inversely, BTG3 was related to the drug resistance to SR16157, Elesclomol, Tamoxifen, Fulvestrant, LDK-378 and Palbociclib. For CMap analysis, we divided the tumor cell lines into high and low expression groups based on the median values of APRO family gene expression, and then calculated the differential expression genes between the high and low expression groups. We selected the top 150 high expression genes and uploaded them to the CMap database to obtain the corresponding compounds for each APRO family genes (Supplementary Table S4). Interestingly, as seen in Table 1, we observed that TOB1 was also positively correlated with drug sensitivity to Elesclomol with a CMap Score > 90. Same predicted targets (font red and box yellow to be prominently presented in the table) were also found for BTG2 and BTG3, nevertheless |CMap Score|< 90, which means extremely low correlation^31^. The reason for the difference in drug target prediction between the two databases may be that each database contains different numbers of compounds.

**Table 2:** CMap study results for APRO family genes.

TOB1		BTG2		BTG3	
Compound	CMap score	Compound	CMap score	Compound	CMap score
**Elesclomol**	**94.4**	Temsirolimus	− 16.68	Tamoxifen	− 41.02
Fulvestrant	− 69.9	Fluphenazine	− 74.68	Palbociclib	− 34.39
				Fulvestrant	89.04

Here CMap score > 0 means positively correlation, while CMap score < 0 means negatively correlation.

### Relationship between expression of APRO family and TMB/MSI

**Figure 9:**
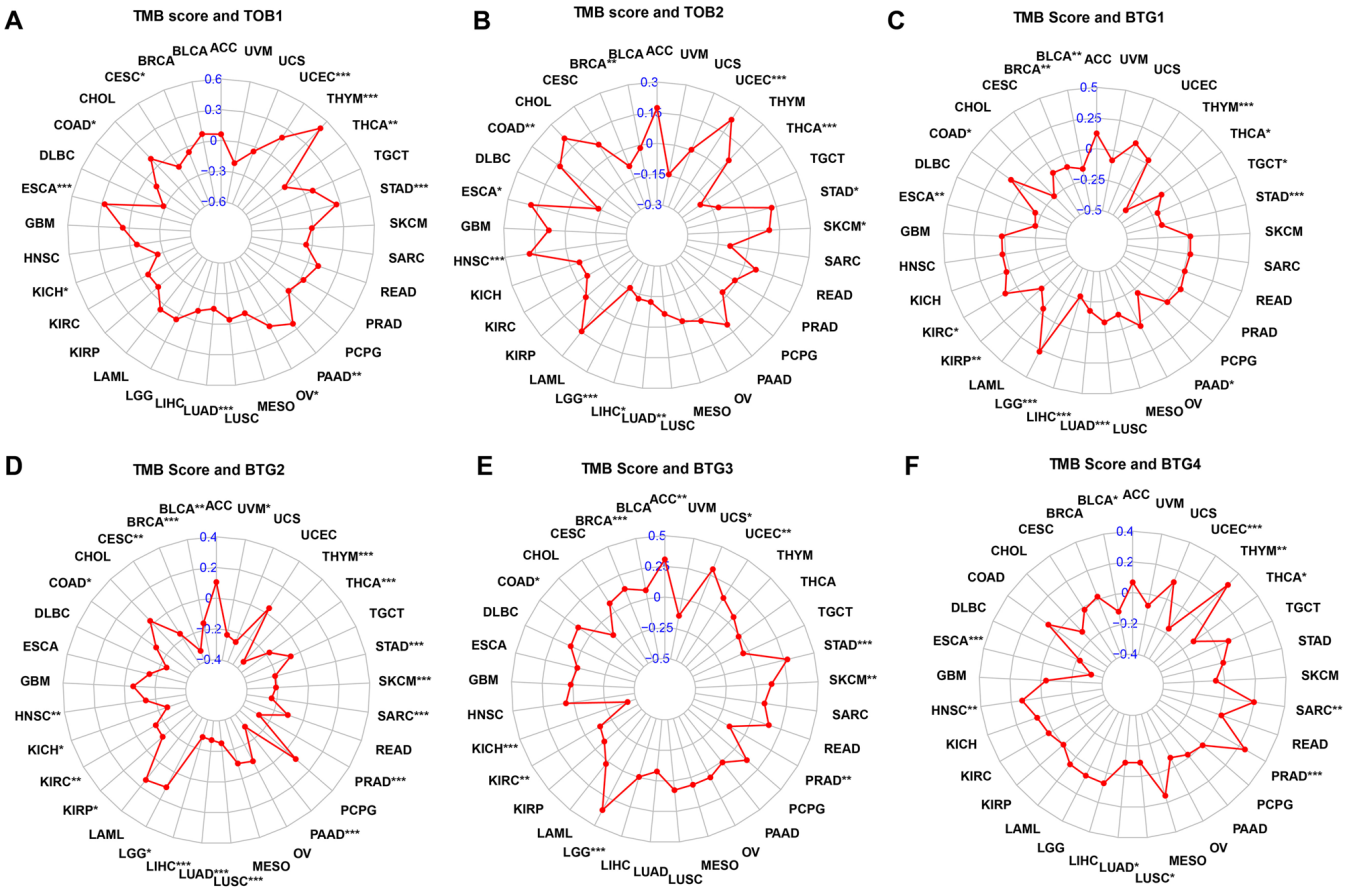
Spearman correlation analysis of TMB score of 33 cancer types and APRO family genes expression distribution. (**A**) TOB1; (B) TOB2; (**C**) BTG1; (**D**) BTG2; (**E**) BTG3; (**F**) BTG4. (***P < 0.001; **P < 0.01; *P < 0.05). TMB, tumor mutation burden.

**Figure 10:**
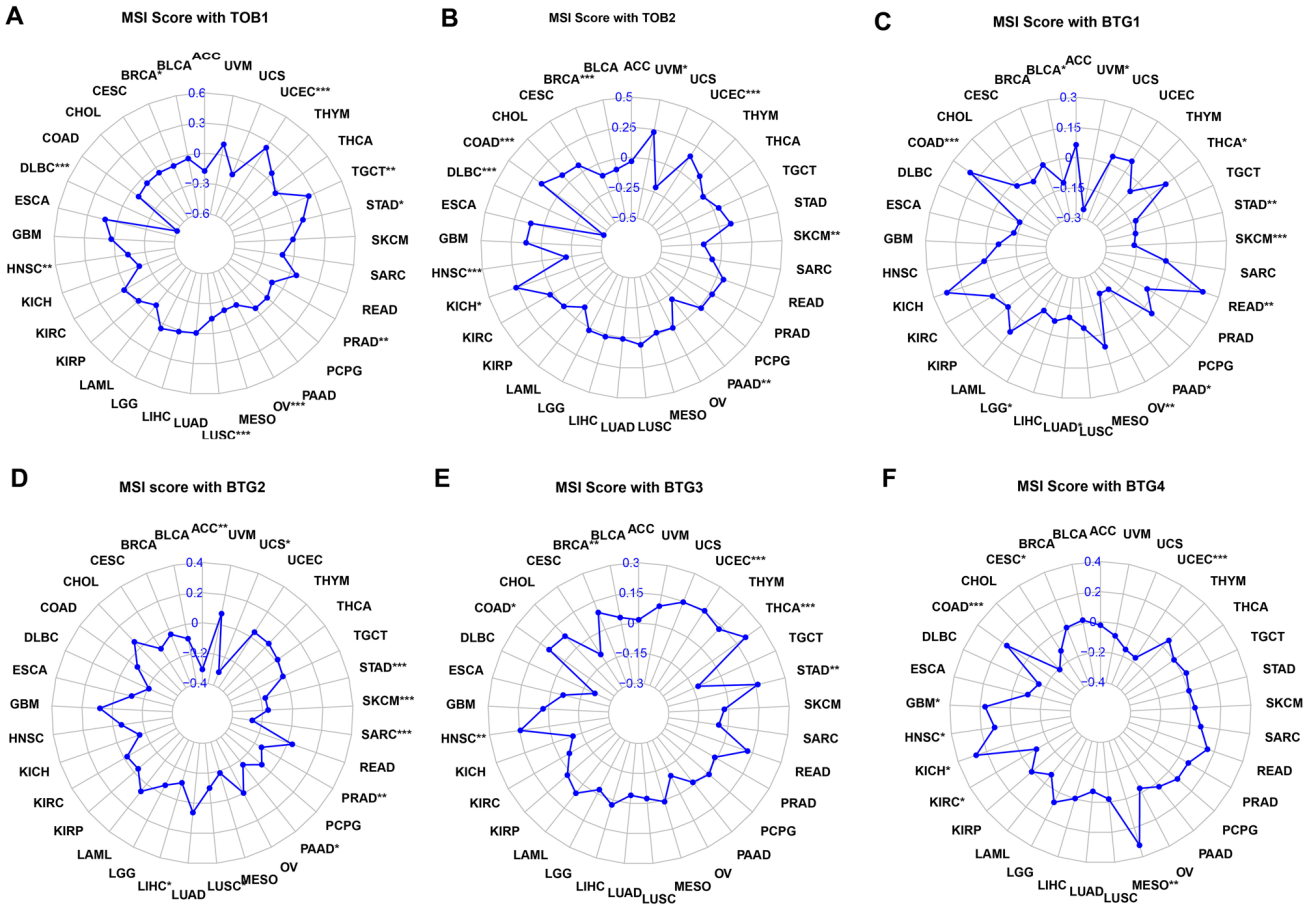
Spearman correlation analysis of MSI score of 33 cancer types and APRO family genes expression distribution. (**A**) TOB1; (**B**) TOB2; (**C**) BTG1; (**D**) BTG2; (**E**) BTG3; (**F**) BTG4. (***P < 0.001; **P < 0.01; *P < 0.05). MSI, microsatellite instability.

We then used the R package “ggstatsplot” to analyze whether the APRO family expression linked to the level of TMB and MSI in TCGA database. The results revealed that TOB1 and TMB for CESC, COAD, KICH, LUAD and THCA, showed a negative association, but we found a positive correlation for ESCA, OV, PAAD, STAD, THYM and UCEC (Fig. 9A). In addition, we found a negative correlation between TOB2 expression and TMB for BRCA, LGG, LIHC, LUAD and THCA (Fig. 9B). The TMB in BTG1 showed a negative correlation with BLCA, BRCA, ESCA, KIRP, LIHC, LUAD, PAAD, STAD, TGCT, THCA and THYM. However, we found that BTG1 had a positive association with COAD and LGG (Fig. 9C). More specifically, the BTG2 expression displayed the association with BLCA, BRCA, CESC, COAD, HNSC, KICH, KIRC, KIRP, LGG, LIHC, LUAD, LUSC, PAAD, PRAD, SARC SKCM, STAD, THCA and THYM in TMB (Fig. 9D). For the BTG3 in TMB associated with ACC, BRCA, COAD, KICH, KIRC, LGG, PRAD, SKCM, STAD, UCEC and UCS (Fig. 9E). In TMB, BTG4 expression associated with BLCA, ESCA, HNSC, LUAD, LUSC, PRAD, SARC, THCA, THYM and UCEC (Fig. 9F). As for the relationship between APRO family and MSI, TOB1 expression was negatively correlated with MSI of BLCA, DLBC, HNSC, LUSC, OV and PRAD (Fig. 10A), but was positively correlated with that of STAD, TGCT and UCEC. The TOB2 expression was negatively correlated with MSI of BRCA, DLBC, HNSC, PAAD, SKCM, but we found TOB2 had a positive correlation with COAD, KICH, UCEC and UVM (Fig. 10B). The BTG1 expression was correlated with MSI of BLCA, COAD, LGG, LUAD, OV, PAAD, READ, SKCM, STAD, THCA and UVM (Fig. 10C). More specifically, the BTG2 expression displayed the association with ACC, LIHC, LUSC, PAAD, PRAD, SARC SKCM, STAD and UCS in MSI (Fig. 10D). The BTG3 in MSI associated with BRCA, COAD, HNSC, STAD, THCA, UCEC (Fig. 10E). In MSI, BTG4 expression was associated with CESC, COAD, GBM, HNSC, KICH, KIRC, MESO and UCEC (Fig. 10F).

### Validation of the expression of APRO family genes in HNSC cells

**Figure 11:**
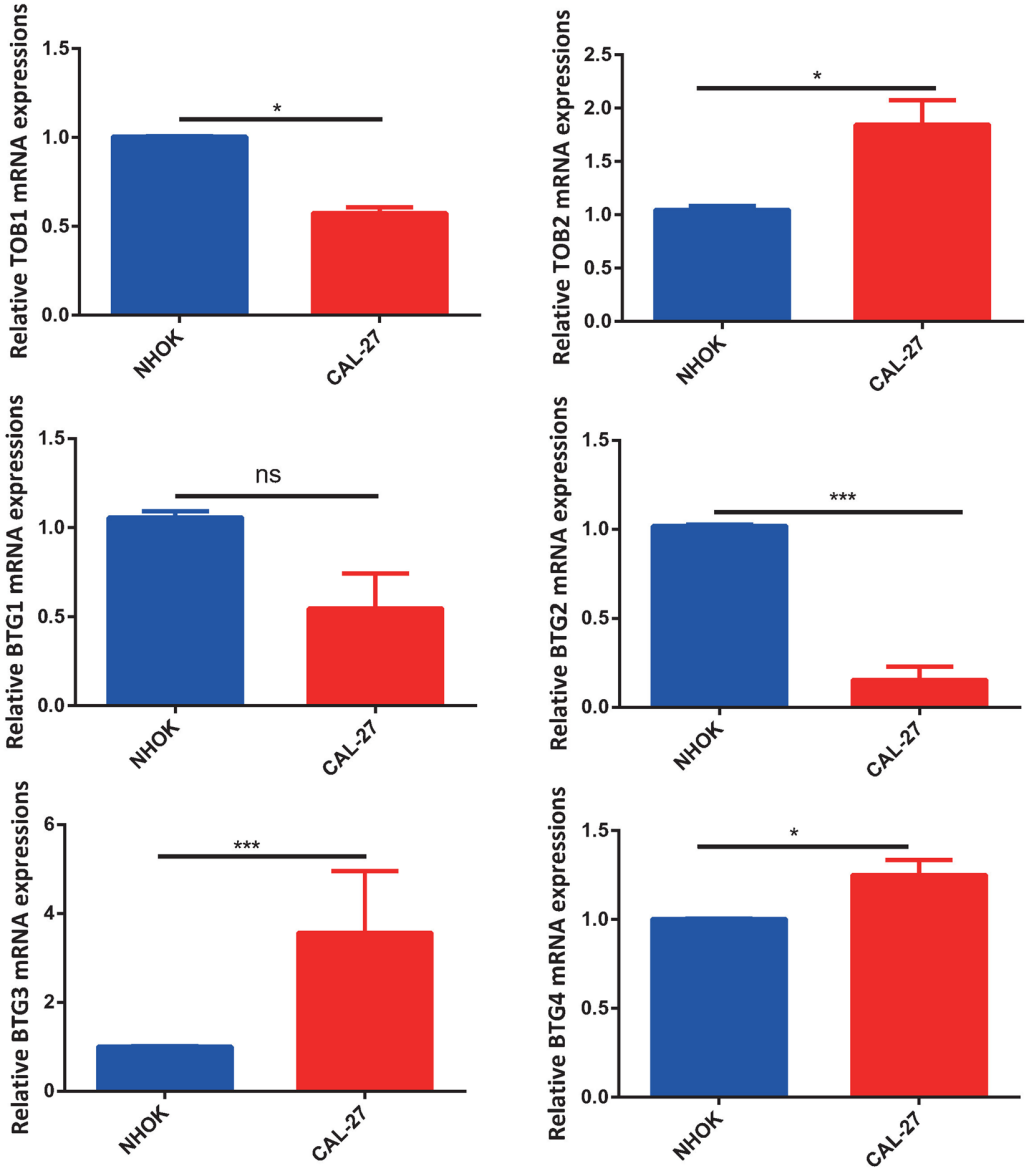
qRT-PCR validation the expression of APRO family genes in HNSC cells. N = 3, the significance of the difference was marked with *, *P < 0.05, ***P < 0.001. The results are presented as mean ± SEM.

Based on the methylation results of bioinformatics analysis, we found that low expression of the hyper-methylated BTG2 gene in HNSC. Therefore, we selected this cancer related cell lines for qRT-PCR validation. We found that BTG2 were significantly low expressed in HNSC cell lines, which was in consistent with bioinformatics analysis result. Additionally, TOB1 and BTG1 were also under-expressed in CAL-27 cells, while TOB2, BTG3 and BTG4 were highly expressed in CAL-27 cells (Fig. 11).

## Discussion

Tumor invasion as a significant issue is a hot topic in current research^32^. As a new class of important factors that can effectively regulate the invasiveness of cancer cells, different APRO family members have different functions in tumor cells. For example, the low TOB1 expression activates gastric cancer progression by inhibiting Smad4- and activating β‑catenin-mediated signaling pathways^33^. BTG1 inhibits proliferation, migration, and invasion of endometrial carcinom by the epithelial-to-mesenchymal transition process^34^. With further investigation of the relationship between APRO family members and tumors, the APRO family may become a basis for tumor diagnosis, and APRO family may be a new index for clinical treatment and prognostic assessment.

Drug sensitivity is an important influencing factor for the effectiveness of drug therapy. A previous research revealed that elevated BTG2 could enhance the responsiveness to Tamoxifen in estrogen receptor-positive breast cancer (BC) patients^35^. To our knowledge, this is the first time that we thoroughly explored the drug sensitivity of some APRO family members. We found that TOB1 and BTG2 exhibited drug resistance to several drugs. Interestingly, both CellMiner and CMap websites revealed that highly expressed TOB1 was sensitive to Elesclomol, an oxidative stress/apoptosis regulator. To our knowledge, TOB1 has been reported to induce apoptosis of cancer cell through kin ds of pathways, such as depressing AKT/mTOR signaling pathway in GC^10^, JNK and p38 pathways in BC^36^. While, elevated levels of BTG3 was related to the drug resistance to Elesclomol. These findings could be applied for drug selection targeting APRO family in the future. What’s more, we also analyzed the tumor stemness score (tRNAss and DNAss). A study showed that cancer stem cells function similarly to the self-renewal of stem cells^37^. The higher stemness score, the stronger biological activity and weaker tumor dedifferentiation ability in tumor stem cells^38^. We observed that most APRO family genes were negatively associated with stemness score, indicating that the expression of APRO family genes could predict the outcomes of stem cell-associated treatment. In fact, the research on stem cell related score (tRNAss and DNAss) and drug sensitivity is rare, we only found one study revealed a stemness-relevant prognostic gene signature on drug sensitivity prediction^39^. The connections between stemness score and drug sensitivity may be a promising research.

We used bioinformatics methods to analyze the characteristics of TOB1, TOB2, BTG1, BTG2, BTG3 and BTG4 in pan-cancer. And based on DNA methylation analysis, we also confirmed the expression of these APRO family genes in HNSC. Unfortunately, although based on DNA methylation prediction, we confirmed the expression of these genes in HNSC cell lines, which was almost consistent with the expression data from TCGA database. The studies about the function of these genes in HNSC cell lines were rarely involved. Nonetheless, massive reviews and researches have revealed the role of APRO family genes on cell growth, migration, as well as invasion in other tumors^2^. For example, TOB1 was up-regulated in colon cancer and BC, indicating a poor prognosis, which was in line with other studies^4, 36^, and elevated TOB1 was demonstrated to promote proliferation in colon cancer through a Wnt positive feedback loop^4^. The study of Ikematsu et al.^40^ showed that TOB2 interacted with Caf1 involved in cell cycle regulation. Moreover, some studies elucidated that BTG1 can be used as a potential prognostic biomarker for endometrial carcinoma^34^ pancreatic ductal adenocarcinoma^3^, colorectal cancer^41^. In consistent with our analysis, overexpressed BTG2 was also found in LUAD^42^, renal cell carcinoma^43^, bladder cancer^44^ and hepatocellular carcinoma^45^. Up-regulated BTG2 could facilitate the apoptosis in non-small cell lung cancer and strengthen the effect of radio-sensitivity^46^. An in vitro experiment had illustrated the inhibitory effect of raised BTG2 on the development of human renal carcinoma cells^47^. In colorectal cancer, BTG3 low expression might strengthen the aggressive behavior^48^. A newly published paper uncovered that BTG4 is a p53 target gene, and overexpressed BTG4 could depress cell growth and induces apoptosis in lung and colorectal cancers^49^. On the foundation of above study background, in the future, the mechanism of APRO family members in occurrence and development of HNSC is worth deep research.

MSI testing and TMB are genomic biomarkers employed to distinguish patients that are possibly to benefit from immune checkpoint inhibitors (ICIs)^50^. MSI is usually caused by mismatch repair deficient (dMMR), which accumulates high levels of mutations and produces new antigens, resulting in higher sensitivity to PD-1/PD-L1 antibodies. The results of whole exon sequencing showed that MSI tumors had more somatic mutations, and a higher rate of somatic mutations was associated with longer PFS, indicating that dMMR based anti PD-1 therapy would be a breakthrough in immunotherapy^51^. In May 2017, FDA approved Pabolizumab for the treatment of microsatellite instability high (MSI-H) solid tumors such as CRC^52^, endometrial cancer^53^ and breast cancer^54^. Also, high TMB is served as an immunotherapy biomarker for ICIs^55^. Here, we found that APRO family members had close connections with MSI and TMB related cancers. Therefore, we believed that APRO family members which were positively correlated with both TMB and MSI related cancers may also be potential targets for ICIs treatment in the future.

Thorsson et al.^24^ found that the C1–C6 immune subtype exists in malignant tumors, forming a specific immune environment, which plays a crucial role in cancer prognosis to predict disease outcomes. Interestingly, we observed that APRO (TOB1, TOB2, BTG1, BTG2, BTG3 and BTG4) family genes were associated with immune invasion subtypes in TME, and BTG1 and BTG2 were highly expressed in C6, however, TOB2 had a high expression in C5. StromalScore and ImmunoScore were used to analyze the correlation between APRO (TOB1, TOB2, BTG1, BTG2, BTG3 and BTG4) expression and tumor purity and tumor microenvironment characteristics.

However, our research also has limitations. The specific role of APRO family members in regulating the development and progression of pan-cancer requires more exploration and research^56^. Further experiments using in vivo, in vitro models as well as clinical samples to are needed to confirm our conclusions. And we plan to further explore the APRO family (TOB1, TOB2, BTG1, BTG2, BTG3 and BTG4) in HNSC tumors through molecular and animal experiments.

## Conclusion

Collectively, the present research provided holistic information to analyze transcription profiles of APRO family members as well as predicted their prognostic values in various cancer subtypes through survival analysis, immune infiltration and immunotherapy etc., in an attempt to offer valuable insights in exploring cancer associations with the APRO family genes. Altogether, carcinostatic APRO family genes could serve as specific biomarkers and potential prognosticators in different cancers.

### Supplementary Information

Supplementary Information.

Supplementary Figure 1.

Supplementary Information.

Supplementary Figure 2.

Supplementary Table 4.

## Data Availability

Publicly available datasets were analyzed in this study. This data can be found here: https://portal.gdc.cancer.gov/repository.
