# Supplementary material for: A pan-cancer analysis of anti-proliferative protein family genes for therapeutic targets in cancer
Source: Sci Rep. 2023 Dec 7;13:21607. doi: 10.1038/s41598-023-48961-1 (PMC10703880; doi:10.1038/s41598-023-48961-1)
Supplement: Supplementary file 1 — Supplementary Information. [file 41598_2023_48961_MOESM1_ESM.docx]

**Supplementary Figure S2 APRO family gene expression differed in certain cancer and normal tissues through TIMER2 analysis.**

**Supplementary Table S4 CAMP analysis for gene target prediction.**
