# Supplementary material for: A pan-cancer analysis of anti-proliferative protein family genes for therapeutic targets in cancer
Source: Sci Rep. 2023 Dec 7;13:21607. doi: 10.1038/s41598-023-48961-1 (PMC10703880; doi:10.1038/s41598-023-48961-1)
Supplement: Supplementary file 3 — Supplementary Information. [file 41598_2023_48961_MOESM3_ESM.docx]

Supplement Figure 1 Survival analysis of APRO family genes


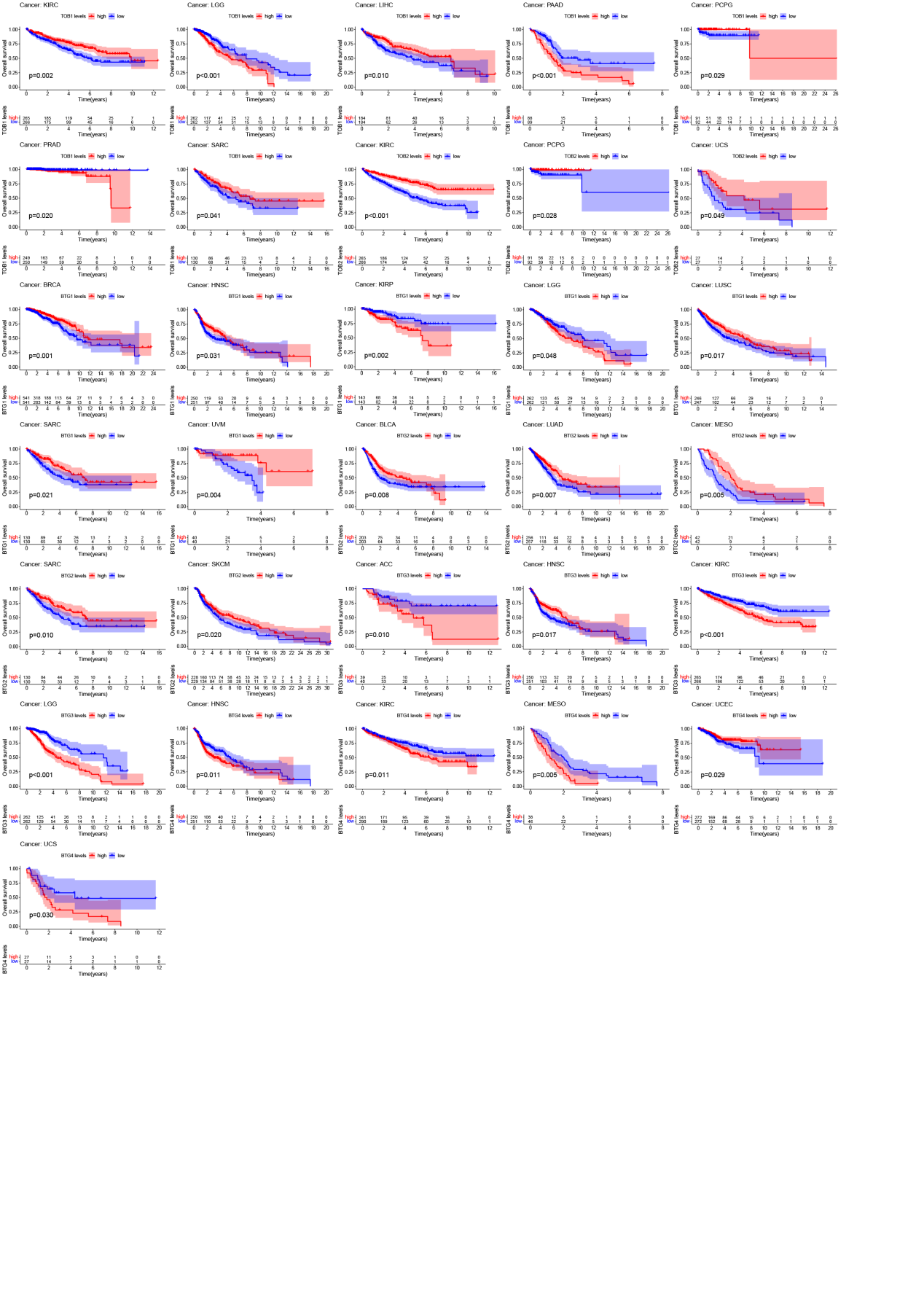


Supplement Figure 1 Survival analysis of APRO family genes.

Table S1. Multivarite test results for APRO genes with pan cancer OS

|  | TOB1 | | | | TOB2 | | | | BTG1 | | | |
| --- | --- | --- | --- | --- | --- | --- | --- | --- | --- | --- | --- | --- |
| cancer | HR | HR.95L | HR.95H | pvalue | HR | HR.95L | HR.95H | pvalue | HR | HR.95L | HR.95H | pvalue |
| ACC | 1.197 | 0.861 | 1.664 | 0.286 | 1.669 | 0.989 | 2.817 | 0.055 | 1.287 | 0.715 | 2.319 | 0.400 |
| BLCA | 0.900 | 0.790 | 1.024 | 0.111 | 0.891 | 0.696 | 1.141 | 0.360 | 0.890 | 0.736 | 1.075 | 0.225 |
| BRCA | 0.964 | 0.832 | 1.118 | 0.630 | 0.770 | 0.547 | 1.084 | 0.135 | 0.732 | 0.581 | 0.921 | 0.008 |
| CESC | 1.041 | 0.769 | 1.410 | 0.796 | 1.355 | 0.864 | 2.125 | 0.186 | 0.947 | 0.685 | 1.309 | 0.743 |
| CHOL | 1.296 | 0.767 | 2.192 | 0.333 | 1.282 | 0.514 | 3.195 | 0.594 | 0.729 | 0.371 | 1.434 | 0.360 |
| COAD | 1.051 | 0.750 | 1.472 | 0.774 | 1.774 | 1.042 | 3.022 | 0.035 | 0.952 | 0.657 | 1.380 | 0.796 |
| DLBC | 2.142 | 0.819 | 5.603 | 0.121 | 1.395 | 0.568 | 3.425 | 0.467 | 1.967 | 0.726 | 5.330 | 0.183 |
| ESCA | 1.138 | 0.859 | 1.508 | 0.367 | 0.787 | 0.462 | 1.338 | 0.376 | 1.041 | 0.716 | 1.515 | 0.832 |
| GBM | 1.131 | 0.829 | 1.542 | 0.438 | 0.753 | 0.577 | 0.981 | 0.036 | 1.198 | 0.789 | 1.819 | 0.397 |
| HNSC | 0.994 | 0.819 | 1.206 | 0.953 | 1.121 | 0.852 | 1.476 | 0.414 | 0.842 | 0.705 | 1.007 | 0.059 |
| KICH | 0.623 | 0.171 | 2.271 | 0.473 | 0.418 | 0.137 | 1.272 | 0.125 | 1.421 | 0.367 | 5.506 | 0.611 |
| KIRC | 0.690 | 0.551 | 0.865 | 0.001 | 0.525 | 0.409 | 0.674 | 0.000 | 1.204 | 0.910 | 1.594 | 0.193 |
| KIRP | 0.947 | 0.667 | 1.344 | 0.760 | 0.979 | 0.573 | 1.670 | 0.937 | 2.838 | 1.761 | 4.575 | 0.000 |
| LAML | 1.060 | 0.774 | 1.452 | 0.715 | 1.139 | 0.720 | 1.803 | 0.578 | 0.939 | 0.726 | 1.213 | 0.629 |
| LGG | 1.869 | 1.369 | 2.553 | 0.000 | 0.619 | 0.474 | 0.810 | 0.000 | 1.247 | 0.901 | 1.725 | 0.184 |
| LIHC | 0.965 | 0.827 | 1.127 | 0.655 | 1.062 | 0.833 | 1.353 | 0.626 | 1.064 | 0.874 | 1.295 | 0.538 |
| LUAD | 1.035 | 0.879 | 1.220 | 0.677 | 1.105 | 0.837 | 1.459 | 0.481 | 1.040 | 0.847 | 1.276 | 0.709 |
| LUSC | 1.078 | 0.860 | 1.352 | 0.516 | 0.914 | 0.682 | 1.224 | 0.545 | 0.865 | 0.709 | 1.054 | 0.151 |
| MESO | 0.742 | 0.591 | 0.930 | 0.010 | 0.939 | 0.533 | 1.654 | 0.828 | 0.988 | 0.700 | 1.394 | 0.943 |
| OV | 1.030 | 0.853 | 1.243 | 0.760 | 1.123 | 0.883 | 1.428 | 0.344 | 0.883 | 0.699 | 1.115 | 0.296 |
| PAAD | 1.485 | 1.143 | 1.929 | 0.003 | 1.703 | 1.060 | 2.737 | 0.028 | 0.974 | 0.709 | 1.337 | 0.869 |
| PCPG | 0.404 | 0.171 | 0.954 | 0.039 | 0.360 | 0.121 | 1.069 | 0.066 | 0.333 | 0.112 | 0.991 | 0.048 |
| PRAD | 2.798 | 1.062 | 7.370 | 0.037 | 1.762 | 0.479 | 6.486 | 0.394 | 0.753 | 0.233 | 2.436 | 0.636 |
| READ | 1.000 | 0.542 | 1.847 | 0.999 | 0.519 | 0.203 | 1.330 | 0.172 | 1.846 | 0.969 | 3.519 | 0.062 |
| SARC | 0.808 | 0.658 | 0.993 | 0.042 | 0.912 | 0.660 | 1.261 | 0.576 | 0.803 | 0.633 | 1.019 | 0.071 |
| SKCM | 0.943 | 0.803 | 1.108 | 0.478 | 1.196 | 0.917 | 1.559 | 0.187 | 0.773 | 0.649 | 0.921 | 0.004 |
| STAD | 0.899 | 0.722 | 1.119 | 0.341 | 0.788 | 0.579 | 1.072 | 0.129 | 1.058 | 0.811 | 1.380 | 0.678 |
| TGCT | 1.304 | 0.259 | 6.574 | 0.748 | 4.328 | 0.204 | 91.875 | 0.347 | 2.190 | 0.492 | 9.760 | 0.304 |
| THCA | 1.394 | 0.802 | 2.424 | 0.239 | 1.363 | 0.502 | 3.700 | 0.543 | 1.812 | 0.646 | 5.082 | 0.258 |
| THYM | 1.864 | 0.892 | 3.893 | 0.098 | 1.021 | 0.272 | 3.835 | 0.975 | 1.067 | 0.494 | 2.303 | 0.869 |
| UCEC | 1.308 | 1.025 | 1.668 | 0.031 | 0.738 | 0.511 | 1.065 | 0.104 | 0.791 | 0.574 | 1.090 | 0.152 |
| UCS | 0.876 | 0.567 | 1.353 | 0.550 | 0.793 | 0.436 | 1.443 | 0.448 | 0.907 | 0.482 | 1.705 | 0.762 |
| UVM | 1.948 | 1.082 | 3.507 | 0.026 | 1.498 | 0.641 | 3.499 | 0.351 | 0.279 | 0.148 | 0.525 | 0.000 |

|  | BTG2 | | | | BTG3 | | | | BTG4 | | | |
| --- | --- | --- | --- | --- | --- | --- | --- | --- | --- | --- | --- | --- |
| cancer | HR | HR.95L | HR.95H | pvalue | HR | HR.95L | HR.95H | pvalue | HR | HR.95L | HR.95H | pvalue |
| ACC | 1.391 | 0.993 | 1.948 | 0.055 | 1.586 | 1.106 | 2.275 | 0.012 | 0.693 | 0.002 | 228.85 | 0.901 |
| BLCA | 0.923 | 0.833 | 1.024 | 0.131 | 1.017 | 0.831 | 1.244 | 0.872 | 1.953 | 0.207 | 18.421 | 0.559 |
| BRCA | 0.843 | 0.748 | 0.950 | 0.005 | 1.003 | 0.851 | 1.182 | 0.975 | 0.505 | 0.018 | 14.306 | 0.689 |
| CESC | 0.860 | 0.674 | 1.097 | 0.224 | 0.777 | 0.548 | 1.102 | 0.158 | 0.558 | 0.0140 | 22.330 | 0.757 |
| CHOL | 0.877 | 0.550 | 1.399 | 0.583 | 1.079 | 0.443 | 2.628 | 0.868 | 0.374 | 8.41E-08 | 1658874 | 0.900 |
| COAD | 1.058 | 0.803 | 1.395 | 0.689 | 0.974 | 0.690 | 1.375 | 0.881 | 3.076 | 0.0460 | 205.79 | 0.600 |
| DLBC | 1.239 | 0.601 | 2.552 | 0.561 | 0.896 | 0.209 | 3.845 | 0.882 | 92.971 | 0.0002 | 56744899.2 | 0.505 |
| ESCA | 1.338 | 1.047 | 1.709 | 0.020 | 1.009 | 0.671 | 1.519 | 0.964 | 0.033 | 0.0003 | 2.943 | 0.137 |
| GBM | 0.945 | 0.768 | 1.164 | 0.597 | 0.955 | 0.686 | 1.328 | 0.783 | 2.901 | 0.165944925 | 50.69744469 | 0.466 |
| HNSC | 1.019 | 0.888 | 1.170 | 0.786 | 0.741 | 0.611 | 0.898 | 0.002 | 1.188 | 0.438 | 3.224 | 0.735 |
| KICH | 0.348 | 0.133 | 0.915 | 0.032 | 0.640 | 0.330 | 1.240 | 0.186 | 1.73E+14 | 1.486 | 2.02E+28 | 0.047 |
| KIRC | 0.726 | 0.594 | 0.887 | 0.002 | 1.374 | 1.138 | 1.660 | 0.001 | 6.187 | 1.425 | 26.855 | 0.015 |
| KIRP | 1.182 | 0.854 | 1.637 | 0.313 | 1.158 | 0.892 | 1.504 | 0.270 | 0.000 | 3.01E-10 | 668.73 | 0.288 |
| LAML | 1.139 | 0.899 | 1.443 | 0.280 | 1.146 | 0.894 | 1.470 | 0.281 | 0.018 | 1.34E-07 | 2445.2 | 0.506 |
| LGG | 0.975 | 0.784 | 1.212 | 0.818 | 2.473 | 1.899 | 3.220 | 0.000 | 25.054 | 3.187 | 196.924 | 0.002 |
| LIHC | 1.063 | 0.918 | 1.231 | 0.414 | 1.387 | 1.074 | 1.791 | 0.012 | 172046.531 | 2.780 | 10648503435 | 0.032 |
| LUAD | 0.798 | 0.704 | 0.906 | 0.000 | 0.986 | 0.808 | 1.203 | 0.889 | 0.666 | 0.273 | 1.621 | 0.370 |
| LUSC | 1.097 | 0.929 | 1.296 | 0.276 | 0.835 | 0.666 | 1.048 | 0.120 | 0.580 | 0.322 | 1.044 | 0.070 |
| MESO | 0.750 | 0.593 | 0.948 | 0.016 | 1.351 | 0.827 | 2.206 | 0.229 | 172.687 | 1.613 | 18484 | 0.031 |
| OV | 1.006 | 0.881 | 1.150 | 0.925 | 0.918 | 0.803 | 1.050 | 0.213 | 1.235 | 0.398 | 3.839 | 0.715 |
| PAAD | 0.998 | 0.789 | 1.264 | 0.988 | 1.031 | 0.712 | 1.491 | 0.872 | 20.837 | 0.001 | 540570 | 0.558 |
| PCPG | 0.737 | 0.454 | 1.194 | 0.215 | 2.973 | 0.694 | 12.733 | 0.142 | 0.000 | 6.27E-50 | 8.03E+22 | 0.475 |
| PRAD | 0.608 | 0.335 | 1.104 | 0.102 | 0.646 | 0.263 | 1.584 | 0.339 | 0.902 | 6.12E-15 | 1.33E+14 | 0.995 |
| READ | 1.127 | 0.756 | 1.679 | 0.558 | 0.978 | 0.476 | 2.007 | 0.951 | 0.000 | 2.06E-18 | 650385162 | 0.512 |
| SARC | 0.709 | 0.596 | 0.842 | 0.000 | 1.017 | 0.821 | 1.259 | 0.879 | 0.553 | 0.186 | 1.645 | 0.287 |
| SKCM | 0.826 | 0.730 | 0.935 | 0.003 | 1.069 | 0.914 | 1.250 | 0.403 | 1.586 | 0.463 | 5.433 | 0.463 |
| STAD | 1.015 | 0.849 | 1.214 | 0.870 | 0.886 | 0.681 | 1.154 | 0.370 | 2.294 | 0.313 | 16.809 | 0.414 |
| TGCT | 1.379 | 0.474 | 4.012 | 0.555 | 0.763 | 0.134 | 4.357 | 0.761 | 0.028 | 6.69E-08 | 12117 | 0.590 |
| THCA | 0.876 | 0.490 | 1.567 | 0.655 | 0.721 | 0.245 | 2.120 | 0.552 | 391796890304 | 243.072 | 6.32E+20 | 0.014 |
| THYM | 0.993 | 0.461 | 2.139 | 0.985 | 1.480 | 0.349 | 6.275 | 0.594 | 25.216 | 0.133 | 4785 | 0.228 |
| UCEC | 0.996 | 0.814 | 1.220 | 0.972 | 0.998 | 0.756 | 1.317 | 0.988 | 0.189 | 0.040 | 0.899 | 0.036 |
| UCS | 0.926 | 0.686 | 1.249 | 0.615 | 1.010 | 0.600 | 1.703 | 0.969 | 70.49 | 0.331 | 15007 | 0.120 |
| UVM | 1.174 | 0.645 | 2.137 | 0.600 | 1.944 | 1.037 | 3.642 | 0.038 | 0.000 | 1.87E-24 | 14839210917 | 0.433 |

Table S2. Summary of association between APRO gene expression and patient overall survival in different cancer (P<0.05), where “poor” means increased expression of APRO family associates with worse survival, while “good” means increased gene expression associates with better survival.

| **Association between LRIG gene expression and patient overall survival** | | | | | | |
| --- | --- | --- | --- | --- | --- | --- |
| Cancer type | TOB1 | TOB2 | BTG1 | BTG2 | BTG3 | BTG4 |
| ACC |  |  |  |  | Poor |  |
| BLCA |  |  |  | Good |  |  |
| BRCA |  |  | Good |  |  |  |
| CESC |  |  |  |  |  |  |
| CHOL |  |  |  |  |  |  |
| COAD |  |  |  |  |  |  |
| DLBC |  |  |  |  |  |  |
| ESCA |  |  |  |  |  |  |
| GBM |  |  |  |  |  |  |
| HNSC |  |  | Good |  | Good | Poor |
| KICH |  |  |  |  |  |  |
| KIRC | Good | Good |  |  | Poor | Poor |
| KIRP |  |  | Poor |  |  |  |
| LAML |  |  |  |  |  |  |
| LGG | Poor |  | Poor |  | Poor |  |
| LIHC | Good |  |  |  |  |  |
| LUAD |  |  |  | Good |  |  |
| LUSC |  |  | Good |  |  |  |
| MESO |  |  |  | Good |  | Poor |
| OV |  |  |  |  |  |  |
| PAAD | Poor |  |  |  |  |  |
| PCPG | Good | Good |  |  |  |  |
| PRAD | Poor |  |  |  |  |  |
| READ |  |  |  |  |  |  |
| SARC | Good |  | Good | Good |  |  |
| SKCM |  |  |  | Good |  |  |
| STAD |  |  |  |  |  |  |
| TGCT |  |  |  |  |  |  |
| THCA |  |  |  |  |  |  |
| THYM |  |  |  |  |  |  |
| UCEC |  |  |  |  |  | Good |
| UCS |  | Good |  |  |  | Poor |
| UVM |  |  | Good |  |  |  |

Supplementary Table S3. Distribution of immune subtypes in pan-cancer

| **TCGA ID** | **Tumor Samples** | **C1** | **C2** | **C3** | **C4** | **C5** | **C6** | **Not Reported** |
| --- | --- | --- | --- | --- | --- | --- | --- | --- |
| ACC | 79 | 2 | 1 | 23 | 48 | 3 | 1 | 1 |
| BLCA | 411 | 161 | 163 | 30 | 42 | 0 | 2 | 13 |
| BRCA | 1104 | 363 | 393 | 194 | 90 | 1 | 38 | 25 |
| CESC | 306 | 75 | 208 | 5 | 10 | 0 | 1 | 7 |
| CHOL | 36 | 8 | 5 | 14 | 7 | 0 | 1 | 1 |
| COAD | 471 | 323 | 87 | 12 | 16 | 0 | 2 | 31 |
| DLBC | 48 | 0 | 0 | 0 | 0 | 0 | 0 | 48 |
| ESCA | 162 | 58 | 73 | 10 | 7 | 2 | 2 | 10 |
| GBM | 168 | 3 | 1 | 0 | 148 | 1 | 0 | 15 |
| HNSC | 502 | 126 | 346 | 13 | 4 | 1 | 4 | 8 |
| KICH | 65 | 2 | 1 | 37 | 12 | 12 | 0 | 1 |
| KIRC | 535 | 11 | 20 | 440 | 26 | 3 | 13 | 22 |
| KIRP | 289 | 11 | 9 | 182 | 67 | 4 | 4 | 12 |
| LAML | 151 | 0 | 0 | 0 | 0 | 0 | 0 | 151 |
| LGG | 529 | 7 | 10 | 13 | 146 | 332 | 1 | 20 |
| LIHC | 374 | 32 | 52 | 133 | 142 | 1 | 2 | 12 |
| LUAD | 526 | 89 | 148 | 171 | 21 | 0 | 27 | 70 |
| LUSC | 501 | 268 | 177 | 19 | 7 | 0 | 15 | 15 |
| MESO | 86 | 30 | 20 | 9 | 13 | 0 | 10 | 4 |
| OV | 379 | 38 | 139 | 2 | 53 | 1 | 1 | 145 |
| PAAD | 178 | 50 | 36 | 43 | 1 | 1 | 19 | 28 |
| PCPG | 183 | 2 | 4 | 106 | 57 | 6 | 2 | 6 |
| PRAD | 499 | 40 | 23 | 292 | 47 | 1 | 0 | 96 |
| READ | 167 | 120 | 18 | 14 | 2 | 1 | 1 | 11 |
| SARC | 263 | 63 | 43 | 42 | 54 | 2 | 19 | 40 |
| SKCM | 471 | 41 | 26 | 15 | 15 | 3 | 3 | 368 |
| STAD | 375 | 116 | 183 | 35 | 11 | 0 | 7 | 23 |
| TGCT | 156 | 43 | 96 | 5 | 4 | 0 | 1 | 7 |
| THCA | 510 | 7 | 18 | 445 | 23 | 2 | 2 | 13 |
| THYM | 119 | 0 | 0 | 0 | 0 | 0 | 0 | 119 |
| UCEC | 548 | 247 | 201 | 52 | 23 | 1 | 1 | 23 |
| UCS | 56 | 38 | 15 | 0 | 2 | 0 | 1 | 0 |
| UVM | 80 | 2 | 1 | 29 | 46 | 2 | 0 | 0 |
