# Supplementary material for: A pan-cancer analysis of anti-proliferative protein family genes for therapeutic targets in cancer
Source: Sci Rep. 2023 Dec 7;13:21607. doi: 10.1038/s41598-023-48961-1 (PMC10703880; doi:10.1038/s41598-023-48961-1)
Supplement: Supplementary file 4 — Supplementary Figure 2. [file 41598_2023_48961_MOESM4_ESM.pdf]

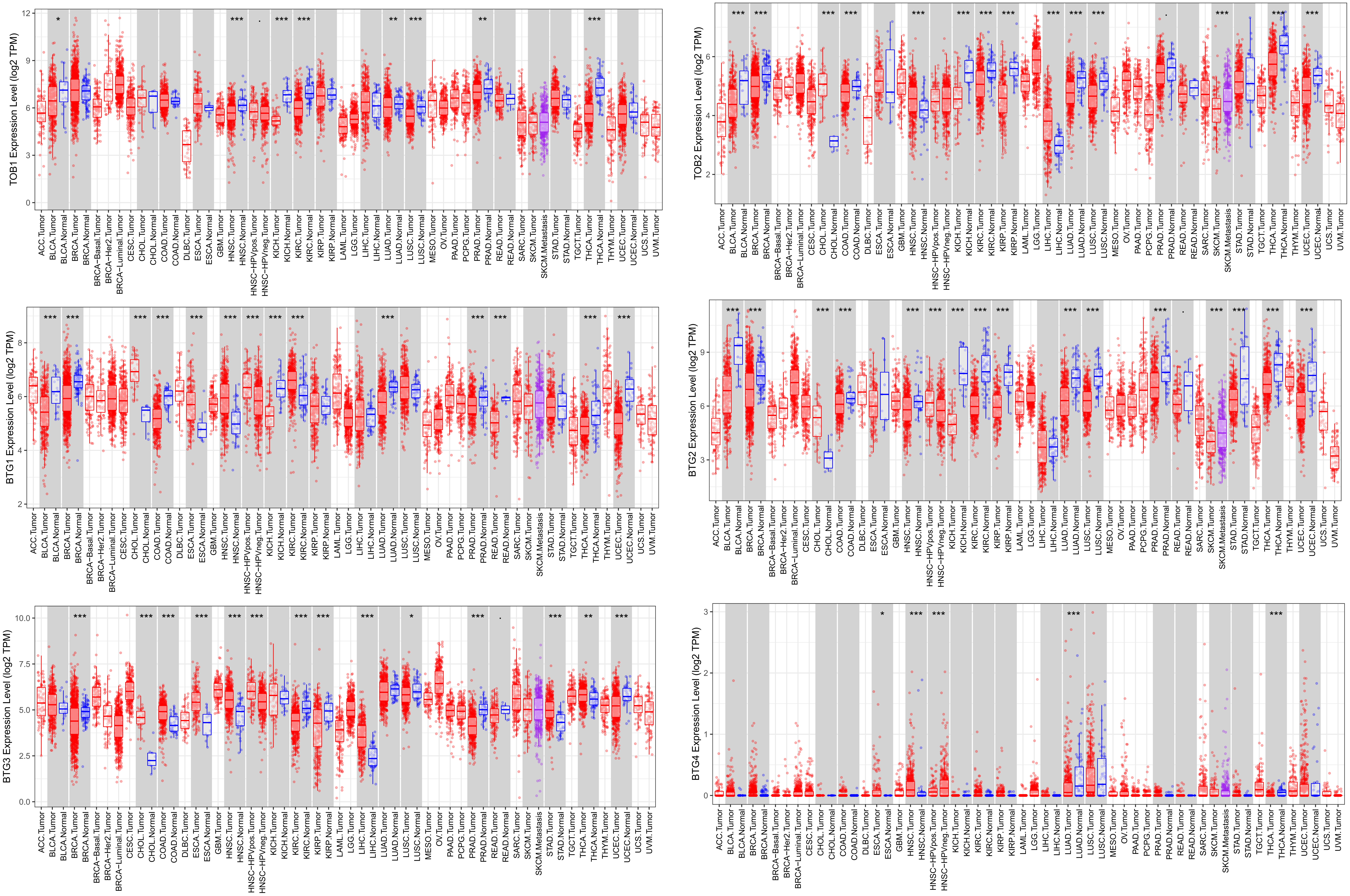
TOB1 Expression Level (log2 TPM)
TOB2 Expression Level (log2 TPM)
BTG1 Expression Level (log2 TPM)
BTG2 Expression Level (log2 TPM)
BTG3 Expression Level (log2 TPM)
BTG4 Expression Level (log2 TPM)
ACC.Tumor
BLCA.Tumor
BLCA.Normal
BRCA.Tumor
BRCA.Normal
BRCA−Basal.Tumor
BRCA−Her2.Tumor
BRCA−Luminal.Tumor
CESC.Tumor
CHOL.Tumor
CHOL.Normal
COAD.Tumor
COAD.Normal
DLBC.Tumor
ESCA.Tumor
ESCA.Normal
GBM.Tumor
HNSC.Tumor
HNSC.Normal
HNSC−HPVpos.Tumor
HNSC−HPVneg.Tumor
KICH.Tumor
KICH.Normal
KIRC.Tumor
KIRC.Normal
KIRP.Tumor
KIRP.Normal
LAML.Tumor
LGG.Tumor
LIHC.Tumor
LIHC.Normal
LUAD.Tumor
LUAD.Normal
LUSC.Tumor
LUSC.Normal
MESO.Tumor
OV.Tumor
PAAD.Tumor
PCPG.Tumor
PRAD.Tumor
PRAD.Normal
READ.Tumor
READ.Normal
SARC.Tumor
SKCM.Tumor
SKCM.Metastasis
STAD.Tumor
STAD.Normal
TGCT.Tumor
THCA.Tumor
THCA.Normal
THYM.Tumor
UCEC.Tumor
UCEC.Normal
UCS.Tumor
UVM.Tumor
